# Drugs for Influenza Treatment: Is There Significant News?

**DOI:** 10.3389/fmed.2019.00109

**Published:** 2019-05-28

**Authors:** Nicola Principi, Barbara Camilloni, Anna Alunno, Ilaria Polinori, Alberto Argentiero, Susanna Esposito

**Affiliations:** ^1^Università degli Studi di Milan, Milan, Italy; ^2^Department of Medicine, Università degli Studi di Perugia, Perugia, Italy; ^3^Department of Surgical and Biomedical Sciences, Pediatric Clinic, Università degli Studi di Perugia, Perugia, Italy

**Keywords:** antiviral drugs, baloxavir marboxil, favipiravir, influenza, monoclonal antibodies, neuraminidase inhibitors

## Abstract

Vaccines remain the best measure to reduce total influenza burden. However, presently available influenza vaccines have some limitations that cause a reduced efficacy compared to immunization practices with other respiratory pathogens. This paper shows the clinical roles of antiviral drugs against influenza that have been licensed in at least one country and the potential roles of compounds that are in development. Several attempts have been made to develop new agents against influenza viruses to overcome the supposed or demonstrated limitations of neuraminidase inhibitors (NAIs). Antibodies against the highly conserved stem region of the haemagglutinin molecule of influenza A viruses and drugs that target different stages of the influenza virus life cycle than NAIs in human cells have been developed and tested. Among these preparations, baloxavir marboxil (BAM), and favipiravir (FP) (i.e., polymerase inhibitors) are the only drugs that have reached the market (the first in Japan and the USA, and the second only in Japan). Other antiviral compounds and monoclonal antibodies are in advanced stage of development, but none of these new drugs and monoclonal antibodies in development have adequate characteristics to substitute for NAIs at present. However, although NAIs remain the drug of choice for influenza treatment, their overuse has to be avoided. Accurate selection of patients for whom treatment is truly needed is required.

## Introduction

Vaccines represent the best way to reduce the influenza impact (1–3). However, influenza viruses vary continuously through antigenic drifts and occasional antigenic shifts that help the virus evade pre-existing immunity (4). Therefore, continuous reformulation of vaccine compositions and annual immunization are needed. In the case of viral shifts, licensed vaccines are completely ineffective, and *ad hoc* vaccine preparations are generally available only several weeks after the emergence and spread of a pandemic influenza virus (5). Finally, the immune responses induced by the influenza vaccines are suboptimal in a number of subjects, especially in younger children and the elderly, who are at risk of severe influenza, which further reduces the protection offered by influenza vaccination (6).

In addition to the intrinsic limitations of influenza vaccines, a second problem limits the vaccine-induced prevention of influenza. Universal immunization against influenza in pediatric age is recommended only in a minority of countries (2). Healthy children and adults frequently are not included in the list of patients for whom official health authorities strongly suggest influenza immunization (7). Moreover, even when vaccines are recommended worldwide, for example, in the elderly, influenza vaccination coverage remains suboptimal (8, 9). The World Health Organization estimates that 5–10% of the global population suffers from influenza every year, 3–5 million people develop severe influenza and 290,000–650,000 people die (10); thus, developing safe and effective alternatives for prophylaxis and treatment is critical.

In this paper, the clinical roles of antiviral drugs against influenza that have been licensed in at least one country will be discussed. Additionally, the potential roles of the anti-influenza compounds in development are evaluated.

## Currently Licensed Anti-influenza Drugs

### Traditional Anti-influenza Virus Drugs

Antiviral drugs have been developed for a long time in an attempt to overcome the abovementioned problems and reduce the influenza-related risks. For years, the adamantane derivatives rimantadine and amantadine and the neuraminidase inhibitors (NAIs) oseltamivir, zanamivir (used worldwide) and, more recently, laninamivir and peramivir (used first in Japan and subsequently in China, Japan, South Korea, and the USA) have been the only drugs licensed for influenza prevention and control. However, these drugs have differences in their pharmacokinetic characteristics, routes of administration and ages of the targeted patients (11).

Starting from the 2004–2005 influenza season, use of adamantane derivatives was no longer recommended, mainly due to the emergence of resistance in most circulating influenza viruses. However, their activity was limited to influenza A viruses, and they showed poor tolerability, which could be considered sufficient reasons *per se* to avoid prescription of these drugs (12). In practice, only NAIs have been prescribed for influenza prevention and treatment since that time period. The emergence of influenza virus strains resistant to NAIs has been reported. Resistance to oseltamivir emerged only during the 2007–2008 and 2008–2009 influenza seasons, with up to 90% of circulating strains exhibiting resistance to this NAI (13–15). Fortunately, the influenza virus strains circulating during the 2009 pandemic and in the following years rarely contained the mutations in the neuraminidase viral surface glycoprotein that conferred resistance to oseltamivir. Localized clusters of oseltamivir-resistant influenza virus have been reported (16) and resistance to NAIs is increasing (17).

However, generally, an influenza virus resistant to oseltamivir is sensitive to the other NAIs, because cross resistance among oseltamivir and other NAIs has not been observed (18, 19). Patients with influenza due to an oseltamivir-resistant virus can be successfully treated with other NAIs, such as zanamivir (15). In patients undergoing treatment, the NAI-resistant viruses are found to be NA subtype–specific and drug-specific (16, 19, 20). These clinically-derived NAI-resistant variants of influenza A viruses of N1 NA subtype most frequently carry H274Y and N294S NA amino acid substitutions. Viruses of N2 NA subtype carry E119Vand R292K NA mutations, and NAI-resistant variants of influenza B viruses harbor R152K and D198N NA mutations. Another important point is that NAI-resistant viruses can emerge either under drug-selection pressure or naturally in the course of influenza virus evolution (without drug intervention). The high prevalence of oseltamivir-resistant A/Brisbane/59/2007(H1N1)-like viruses (subclade 2B) carrying the NA H274Y–resistance marker was reported worldwide during the 2007–2009 influenza seasons (16, 19, 20). Epidemiologic studies did not show evidence of an association between the development of resistance and oseltamivir use. Thus, H274Y NA amino acid substitution occurred naturally, and viruses with this mutation acquired remarkable transmissibility and superior fitness compared to their drug-sensitive counterparts (19, 20).

Moreover, all NAIs have been found to be safe and well tolerated. In particular, oseltamivir, which is the most frequently used NAI, can be administered not only to otherwise healthy adults and subjects with severe underlying disease but also to neonates, younger infants (18) and pregnant women (21) with age- and weight-appropriate doses without significant risks of severe adverse events.

Based on the results of several clinical trials, NAIs have been considered effective for the prevention and control of influenza infection (22, 23). Most available studies in this regard were carried out with oseltamivir and zanamivir, which were marketed before the other NAIs (24–28). Moreover, all of the data collected in younger children were derived from studies in which oseltamivir was used instead of zanamivir, which was licensed for use only in school-age patients. The effectiveness of NAIs has been documented in some meta-analyses, including those by Jefferson et al. (24) and Dobson et al. (25). A global evaluation of all of the studies analyzed in these meta-analyses suggests the conclusion that NAIs can limit the severity and duration of influenza in patients of any age with uncomplicated disease. However, the efficacy is time-dependent, because it is seen mainly in subjects who receive these drugs within the first 48 h of symptom onset. Jefferson et al. (24) and Dobson (25) calculated that administration of oseltamivir in adults with uncomplicated influenza decreased the time to first alleviation of symptoms of influenza-like illness (ILI) by 16.8 h [95% confidence interval [CI], 8.4–25.1] and 17.8 h (95% CI, 27.1 to −9.3), respectively. When only laboratory-confirmed influenza cases were considered, the advantage was slightly higher, because the time to perceived benefits was decreased by 25.2 h (95% CI, 16.0–36.2) (25). Similar results were reported for zanamivir, with the difference in time to alleviation of ILI symptoms reported as 14.4 h (95% CI, 9.36–19.44). No substantial differences were noted in the results obtained in children treated with oseltamivir (24). Regarding prophylaxis, it was calculated that oseltamivir could reduce the absolute risk for laboratory-confirmed influenza among community members and nursing home residents by 3.05% [relative risk [RR] 0.45; 95% CI, 0.30–0.67] and in a household setting by 13.6% (RR 0.20; 95% CI, 0.09–0.44) (21).

However, the available data do not permit firm conclusions regarding the effect of NAIs on severe cases (26–28). Although administration of NAIs was associated with a reduction of mortality during the recent 2009 pandemic (29), the impact on the development of serious influenza complications, hospitalization and mortality was not definitively demonstrated. The results of studies specifically planned to evaluate these problems were conflicting (30). A good example in this regard is given by studies that have measured the prevention of acute otitis media (AOM), which is a common complication of influenza in children. Jefferson et al. (24) did not find any statistically significant effect of oseltamivir on this disease (RR 0.8; 95% CI, 0.62–1.02), whereas Wang et al. (31) reported a statistically significant reduction in the AOM incidence with oseltamivir treatment in children 1–12 years of age (risk difference [RD] −0.09; 95% CI, −0.16 to −0.03). Moreover, in some cases, such as the meta-analysis by Muthuri et al. (32), NAI treatment was shown to be associated with an increased risk of severe outcomes, including pneumonia (OR 2.29; 95% CI, 1.16–4.53). Because laninamivir (33) and peramivir (34) do not seem to have substantially different efficacies compared to that of oseltamivir, these findings explain why the present possibility of treating influenza with NAIs cannot be considered completely satisfactory and the development of new drugs against influenza viruses has been deemed mandatory.

Several new approaches have been attempted to achieve influenza prevention and control. Antibodies against the highly conserved stem region of the haemagglutinin (HA) molecule of influenza A viruses and drugs that target different stages of the influenza virus life cycle than NAIs in human cells have been developed and tested (35). Among these preparations, baloxavir marboxil (BAM) and favipiravir (FP) are the only drugs that have reached the market (the first in Japan and the USA and the second only in Japan).

## Recently-licensed Anti-influenza Virus Drugs

### Baloxavir Marboxil (BAM)

BAM was licensed in 2018 in Japan and the USA for the treatment of uncomplicated influenza in subjects aged ≥ 12 years with influenza clinical manifestations for ≤48 h (36). BAM is a prodrug that is given by mouth and is hydrolysed in the intestine, blood, and liver to form baloxavir acid (BXA), which is the active compound (37). The drug acts by inhibiting the cap-dependent endonuclease activity of the influenza A and B virus polymerase acidic protein (PA) to prevent the so-called cap snatching (i.e., the mechanism used by viruses to deviate the host mRNA transcription system and allow synthesis of viral RNAs) (37). In practice, BMA, in contrast to NAIs that reduce viral release from infected cells (38), inhibits viral replication. *In vitro* studies have shown that this activity is exerted without cytotoxicity even in cells infected with NAI-resistant influenza viruses. Moreover, BMA was shown to be effective not only against influenza viruses that usually infected humans but also against avian subtypes (39).

Phase 1 clinical trials were carried out in a total of 55 healthy subjects aged 20–59 years and were mainly directed to evaluate the safety, tolerability, pharmacokinetics and food effects (40). BAM was well tolerated, as no serious adverse events or deaths were reported. Moreover, treatment-emergent adverse events, such as headache, stomatitis, an increased eosinophil count, and elevation of alanine aminotransferase (ALT) and aspartate aminotransferase (AST), emerged in a very small number of subjects, were mild and resolved spontaneously within a few days. Only one subject had an increased lactate dehydrogenase (LDH) serum concentration that did not disappear during the follow-up period (22 days). Pharmacokinetic data confirmed that the drug could be administered as a single dose because its half-life was very long (more than 70 h). Moreover, even the lowest dose of the drug used in these trials (6 mg of BAM) was associated with high BXA serum concentrations 24 h after the single dose. Finally, food could influence drug metabolism, since BXA concentrations were lower in subjects who were fed or when measured before meal states. However, the levels remained high enough to ensure significant viral inhibition (40).

Phase 2 and 3 studies involving subjects aged 12–64 years confirmed the efficacy and safety of BAM (41). Adverse events, such as diarrhea, bronchitis, nausea, common cold symptoms (nasopharyngitis), and headache, were reported in ~2% of patients and were not considered severe. Moreover, in otherwise healthy subjects with fever and mild to moderate uncomplicated respiratory disease for <48 h, administration of the drug could lead to a reduction of the mean time to alleviation of symptoms of ~50–65 h compared to the 77.7–80 h required for patients who received a placebo. However, the clinical efficacy was not different from that evidenced in patients receiving oseltamivir, although BAM administration was associated with a more rapid decline in the viral load and a shorter duration of infectious virus detection (41). Similar results were obtained with a double-blind, randomized, placebo-, and oseltamivir-controlled trial carried out in patients >12 years old suffering from influenza for <48 h with an underlying disease that was considered a risk factor for influenza-related complications. In this study, a shorter time to improvement of symptoms was also evidenced in the BAM group than in the placebo group (median 73.2 h vs. 102.3 h, *p* < 0.0001), whereas the efficacy of oseltamivir was quite similar (81.0 h, *p* = 0.8347). However, when the efficacy against the different types of influenza viruses was tested, BAM and oseltamivir demonstrated similar efficacies against the A/H3N2 virus, but the new drug was superior to oseltamivir against B viruses (time to symptom improvement 74.6 h vs. 101.6 h; *p* = 0.0251) (41). Moreover, despite the presence of severe underlying diseases, adverse events remained uncommon and did not differ from those found in patients receiving placebo or oseltamivir.

BAM has at least two important advantages over other available anti-influenza virus drugs. It is administered as a single dose by mouth, and it can overcome the problem of influenza viruses resistant to NAIs (39). Oseltamivir is also given by mouth, but to be effective it needs 5 days of administration, which can reduce compliance. Peramivir is given as a single dose by the intravenous route, requires administration by a nurse or a doctor and is more expensive than BAM. Zanamivir and laninamivir are administered by inhalation and are difficult to use in younger children.

However, despite obtaining a Food and Drug Administration (FDA) license, some unsolved problems seem to indicate that the use of BAM in clinical practice as an alternative to NAIs, particularly oseltamivir, for treatment of uncomplicated influenza cases can be debated. First, BAM is more expensive than oseltamivir. The cost of treatment with BAM has been calculated to be ~3 times higher than that of treatment with oseltamivir (42). Moreover, the epidemiological and clinical importance of resistance to BAM is not precisely defined. Before obtaining the license, almost all isolated influenza virus strains were susceptible to BAM. A study carried out in the USA showed that the frequency of genetic mutations (amino acid 38 substitutions for isoleucine in the endonuclease domain of the viral RNA polymerase PA subunit) that caused reduced susceptibility of influenza viruses to BAM was very low during the 2016/2017 and 2017/2018 seasons (43). Moreover, no mutated virus was detected in 2018/2019 before BAM was licensed (43). However, treatment was associated with a rapid and substantial emergence of resistant strains. Some of the studies used to obtain the license evidenced that mutated viruses could be identified in 2.2–9.7% of cases (41). Resistance to BAM was also evidenced in Japan after its introduction in clinical practice. A pediatric study showed that ~20% of treated children developed mutations (44). Recently, this problem was confirmed in strains isolated from adult patients (45). Although the mutated viruses remained sensitive to NAIs and had an impaired replicative capacity *in vitro* (44), patients with mutations shed the viruses and remained symptomatic for a longer period than those without the mutations (41), suggesting a potential negative effect of resistance to BAM.

These findings seem to suggest that emergence of resistance to BAM should be strictly monitored and that the clinical effects of BAM should be more carefully evaluated. Because definitive data in this regard are lacking, the routine use of BAM for treatment of uncomplicated influenza in adults seems premature. On the other hand, development of BAM is not complete. BAM is not licensed for complicated influenza cases. Moreover, data from children, pregnant women and patients at risk are rare or completely lacking, as are data concerning influenza prophylaxis.

### Favipiravir (FP)

FP is a prodrug. FP is a nucleoside analog that after oral ingestion requires intracellular phosphoribosylation to be transformed into its active form, FP ribofuranosyl-5′-triphosphate (FRTP). Similar to BAM, FRTP acts by reducing viral replication through inhibition of the RNA-dependent RNA polymerase (RdRp) of RNA viruses (46). However, its activity is not limited to influenza viruses but instead is extended to several other RNA viruses, including arenaviruses, phleboviruses, hantaviruses, flaviviruses, enteroviruses, respiratory syncytial virus, and noroviruses (47). *In vitro* studies have shown that FP is effective against all of the influenza virus subtypes, including the avian viruses and those that are poorly sensitive or insensitive to NAIs (48–50). In experimental animals, FP was found to be significantly more effective than oseltamivir in preventing influenza or reducing its severity in mice infected with lethal doses of influenza viruses, even when the treatment was started 72 h after infection (51). Moreover, when used in combination with oseltamivir, protection due to the NAI was increased and the treatment efficacy window was extended to 96 h after symptom onset (52).

The safety and tolerability of FP were found to be good, although some data regarding the potential teratogenicity of FP were collected in all animal species assessed (53). Interestingly, the exposure causing teratogenicity was quite similar to that found in humans receiving the dosage of the drug that was useful for influenza treatment. Finally, although *in vitro* studies have shown that viral mutations associated with non-viable viral phenotypes rapidly develop in influenza viruses exposed to this drug, *in vivo* use of FP is only rarely associated with influenza virus mutations (54).

Some phase 2 and 3 studies in adults have been carried out in Japan, the USA and Europe (NCT01068912, NCT02026349, NCT02008344, NCT01728753, and NCT03394209). Generally, the drug was administered by mouth two times per day for 5 days even when different dosages were used. Most studies were completed several months ago, but the results are not available with the exception of those from NCT01068912, which aimed to identify the most effective and best tolerated drug dosage. The reasons for the unavailability of the study results are unknown. However, FP is licensed for use in humans in Japan. Its use is authorized only in patients infected by a novel or re-emerging influenza virus (i.e., a pandemic virus?) that is resistant to all other available influenza drugs. To avoid irrational prescriptions, FP is distributed only upon request by the Minister of Health, Labor and Welfare of Japan. Moreover, the guidelines clearly establish that pregnant women are excluded from use of FP, and females of childbearing potential have to avoid pregnancy in the 7 days following drug use to obtain the complete elimination of the FP concentration from the plasma (55).

## Antiviral Preparations in Advanced Stages of Development

### Pimodivir

Pimodivir is an oral drug that inhibits the polymerase basic protein 2 (PB2) subunit of the influenza A virus polymerase complex. Consequently, it inhibits viral replication (56). *In vitro* and experimental animal studies have shown that pimodivir is effective against influenza A virus, even when strains resistant to NAIs are tested. Compared to oseltamivir, it was more effective in improving body weight and reducing the severity of lung infection. However, its activity can significantly vary according to the influenza A virus subtype, probably as a consequence of differences in the PB2 structure among various A viruses (57). Positive results were also obtained when the drug was tested in humans with uncomplicated influenza, for whom pimodivir reduced viral shedding and influenza symptoms. Generally, adverse events (mainly diarrhea) were mild and spontaneously resolved within a few days (58). These findings were confirmed by a double-blinded phase 2b study involving adults with uncomplicated influenza A in whom different pimodivir dosages were given 2 times per day for 5 days alone or in combination with oseltamivir (59). This study showed that 600 mg of the drug per dose was adequate to obtain the best results. In the treated groups, both the viral load titer and the time to symptom resolution were reduced compared to those of the placebo group. Moreover, combined therapy was more effective than administration of pimodivir alone (59, 60).

However, the use of pimodivir has been associated with the emergence of mutations leading to viruses with a several fold decrease in susceptibility to the drug. In particular, emergence of PB2 substitutions or phenotypic resistance to pimodivir was evidenced in 11 of 172 patients (6.4%) (59, 60). The mutations included S324K/N/R, F325L, S337P, K376N/R, T378S, and N510K (59, 60). Although the mutated viruses were not associated with a deterioration of clinical conditions and the patients did not shed the virus after treatment, this finding deserves attention.

### Monoclonal Antibodies

Studies of natural immune responses to influenza virus infection evidenced that N-linked glycosylation sites in the haemagglutinin stem region of influenza A viruses were relatively well conserved and that antibodies against these sites were effective against a large number of influenza viruses (61–64). Starting from this evidence, a number of monoclonal antibodies targeting the HA conserved regions was developed and tested for the treatment of influenza A.

Preparations identified as MHAA4549A, MEDI8852, and VIS 410 have reached the phase II stage of development. All of these preparations were found to be safe and well tolerated and were able to reduce the peak viral load, duration of viral shedding, and influenza symptom scores compared to those of the placebo group (65–67). Moreover, no mutated virus was evidenced after monoclonal antibody administration (65–67). Compared to those of oseltamivir, all of these preparations have superior pharmacokinetic properties and a longer therapeutic window. The half-life is ~3 weeks, one dose can be sufficient to control infection and efficacy can be obtained even if administration occurs after 48 h from symptom onset (65, 68, 69). However, two clinical trials (NCT02293863 and NCT02603952) in which the efficacy of oseltamivir alone was compared to that of the combination oseltamivir/MHAA4549A or MEDI8852 in adults with influenza A infection revealed that addition of the monoclonal antibody did not add any significant clinical advantage to those offered by the old NAI. When these results are considered together with the fact that the activity is limited to influenza A viruses, this finding seems to be a relevant limitation that may preclude extensive use of monoclonal antibodies. However, a definitive conclusion in this regard can only be drawn when studies in patients with severe influenza illnesses and those at high risk, including children and pregnant women, are performed.

## Conclusions

Table 1 summarizes antiviral drugs against influenza available on the market. Several attempts have been made to develop new agents against influenza viruses that are able to overcome the supposed or demonstrated limitations of NAIs. These drugs, mainly oseltamivir, are discussed in terms of the risk of emergence of resistant strains and doubts regarding their efficacy in severe influenza cases and prevention of bacterial complications or death. Some of the new drugs have been licensed or are in the advanced stages of development. However, none of them has been completely developed, and the lack of data regarding patients for whom oseltamivir is recommended is discussed. Moreover, in some cases, use of the new drugs has been associated with emergence of resistance. In practice, none of these new drugs and monoclonal antibodies that are in development has adequate characteristics to substitute for NAIs at present. However, although NAIs (especially oseltamivir due to its greater ease of administration) remain the drug of choice for influenza treatment, their overuse has to be avoided. Accurate selection of patients for whom treatment is truly needed is necessary. Finally, the limitations of antivirals can be overcome with more extensive use of influenza vaccines.

**Table 1:** Antiviral drugs against influenza available on the market.

**Antiviral drugs**	**Characteristics**
**Adamantane derivatives**	
Rimantadine Amantadine	Prevention of the uncoating of the virus's protective shells, which are the envelope and capsid of influenza virus Activity limited on influenza A virus Poor tolerability Emergence of resistance No longer recommended starting from 2004 to 2005
**Neuraminidase inhibitors (NAIs)**	
Oseltamivir Zanamivir Laninamavir Peramivir	Reduction of viral release from the infected cells Activity on influenza A and B viruses Good tolerability Emergence of resistant strains without cross-resistance between the drugs Laninamavir and peramivir are used only in Japan, China, South Korea, and the USA
**Polymerase inhibitors**	
Baloxavir marboxil Favipiravir	Inhibition of viral replication Activity on influenza A and B viruses Efficacy on NAIs-resistant strains Effective on avian influenza subtypes Good safety and tolerability More expensive Problem of resistance still unknown

## Author Contributions

NP wrote the first draft of the manuscript. BC and AAl gave scientific contributions. IP and AAr performed the literature review. SE co-wrote the manuscript and supervised all activities. All authors approved the final submitted version of the manuscript.

### Conflict of Interest Statement

The authors declare that the research was conducted in the absence of any commercial or financial relationships that could be construed as a potential conflict of interest.
